# Prognostic, Predictive, and Clinical Relevance of DNA Damage Repair Alterations in Biliary Tract Cancers

**DOI:** 10.3390/cancers18132134

**Published:** 2026-07-01

**Authors:** Jawad Tarfouss, Oier Azurmendi Senar, Kosta Stosic, Laurine Verset, Christelle Bouchart, Julie Navez, Jean-Luc Van Laethem, Anne Demols, Tatjana Arsenijevic

**Affiliations:** 1 Laboratory of Experimental Gastroenterology, Université Libre de Bruxelles, Route de Lennik 808, 1070 Brussels, Belgiumjean-luc.vanlaethem@hubruxelles.be (J.-L.V.L.); anne.demols@hubruxelles.be (A.D.); 2 Department of Pathology, Hôpital Universitaire de Bruxelles (H.U.B.) Bordet Erasme, Université Libre de Bruxelles, 1070 Brussels, Belgium; 3 Department of Radiation Oncology, Hôpital Universitaire de Bruxelles (H.U.B.) Bordet Erasme, Université Libre de Bruxelles, 1070 Brussels, Belgium; 4 Department of Hepato-Biliary-Pancreatic Surgery, Hôpital Universitaire de Bruxelles (H.U.B.) Bordet Erasme, Université Libre de Bruxelles, 1070 Brussels, Belgium; 5 Department of Gastroenterology, Hepatology and Digestive Oncology, Hopital Universitaire de Bruxelles (H.U.B.), Université Libre de Bruxelles, 1070 Brussels, Belgium

**Keywords:** biliary tract cancers, DNA damage repair, biomarkers, targeted therapy, clinical trials

## Abstract

Biliary tract cancers (BTCs) comprise a heterogeneous group of malignancies arising from the biliary tree, including cholangiocarcinomas and gallbladder carcinomas. Although their incidence is rising globally in Western countries, these cancers are rare and most often diagnosed at advanced stages. Their molecular heterogeneity and frequent resistance to therapy further contribute to their dismal prognosis. In recent years, molecular profiling studies have led to a better understanding of BTC biology and have opened new therapeutic opportunities, particularly for patients with advanced or metastatic disease. Alterations in DNA damage repair (DDR) genes have been identified in a substantial proportion of BTC cases, with some cohorts observing frequencies approaching ~70%. These genetic alterations play a critical role in tumorigenesis and disease progression and may contribute to treatment resistance. In this article, we discuss the current knowledge on: (1) the main DDR signaling pathways; (2) the prevalence of DDR gene alterations across BTC subtypes; (3) the potential of DDR gene alterations as prognostic and/or predictive biomarkers of treatment response; and (4) the latest advances in DDR-based targeted therapies and ongoing clinical trials in BTC.

## Simple Summary

Biliary tract cancers (BTCs) are rare and aggressive malignancies of the biliary system associated with a very poor prognosis. These tumors are clinically, anatomically, and molecularly heterogeneous, which explains their frequent resistance to conventional treatments. Genomic studies have provided a clearer molecular portrait of BTCs and have reported recurrent alterations in genes involved in DNA damage repair (DDR) machinery. These genetic aberrations contribute to tumorigenesis and metastasis formation, and emerging data suggests that DDR also plays an important role in treatment resistance. This review article summarizes the current knowledge on the major DDR pathways, the frequency of DDR alterations across BTC subtypes, and the significance of these alterations as prognostic and/or predictive biomarkers, as well as therapeutic targets.

## 1. Introduction

Biliary tract cancers (BTCs) comprise a group of highly aggressive tumors that originate from different parts of the biliary system. These include cholangiocarcinomas (CCAs), which arise from the biliary tree, and gallbladder carcinomas (GBCs), which develop in the gallbladder or cystic duct [1,2]. CCAs are further anatomically classified as: (1) intrahepatic CCA (iCCA), which originates from the small or large bile ducts within the liver; (2) perihilar CCA (pCCA), previously called “Klatskin tumors”, arising from the right, left, and/or common hepatic ducts; and (3) distal CCA (dCCA), which develops in the common bile duct (choledochus) [3,4,5]. In some studies, pCCA and dCCA are collectively referred to as extrahepatic CCA (eCCA) [6].

Although BTCs are relatively rare, the incidence of CCA has been steadily increasing, particularly that of iCCA, likely due to the rising prevalence of chronic liver diseases [7,8]. Conversely, the incidence of GBC has been declining, largely because of the routine use of cholecystectomy in Western countries [9]. All BTC subtypes are associated with a very poor prognosis, with 5-year overall survival (OS) rates ranging from only 5% to 20% [10]. Furthermore, according to the World Health Organization and the International Agency for Research on Cancer, global mortality rates from BTC have risen in recent years [11,12].

**Figure 1:**
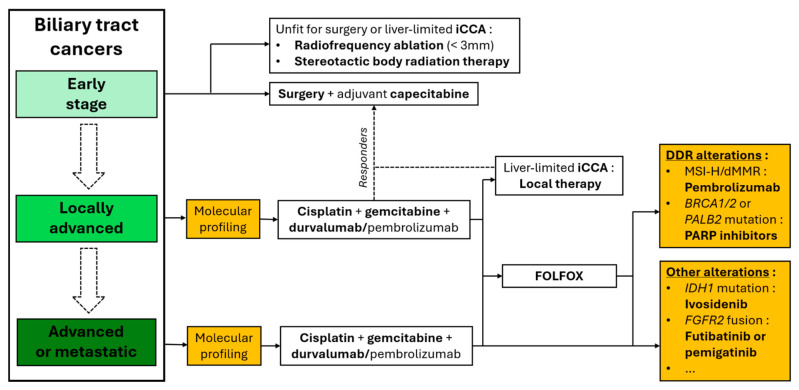
Summary of treatment options for patients with biliary tract cancer according to disease stage. 5-FU: 5-fluorouracil; *BRCA1/2*: *breast cancer 1 or 2*; DDR: DNA damage repair; *FGFR2*: *fibroblast growth factor receptor 2*; FOLFOX: combination of folinic acid + 5-FU + oxaliplatin; iCCA: intrahepatic cholangiocarcinoma; *IDH1*: *isocitrate dehydrogenase 1*; MSI-H/dMMR: high microsatellite instability and deficient mismatch repair; *PALB2*: *partner and localizer of BRCA2*. Adapted from [23].

Most patients with BTC (~70%) are diagnosed at an advanced stage, primarily due to the absence of specific early symptoms [13,14]. Only a small proportion of patients (~30%) are diagnosed at an early stage and are eligible for surgical resection and lymphadenectomy. The type of surgery depends on tumor location and is typically followed by adjuvant chemotherapy with capecitabine in Western countries [15]. At present, radical surgical resection remains the only curative treatment option for BTCs [6]. However, the recurrence rate after surgery is high, reaching up to 70% [16,17,18]. In patients with advanced or unresectable disease, molecular profiling of the tumor is recommended before initiating systemic treatment. The current first-line standard of care (SoC) consists of cisplatin, gemcitabine, and durvalumab or pembrolizumab, two immune checkpoint inhibitors (ICIs) [19,20]. Upon disease progression, patients receive second-line SoC therapy with FOLFOX (combination of folinic acid, 5-fluorouracil (5-FU), and oxaliplatin) and/or molecular-targeted therapies (MTTs) adapted to the tumor’s molecular profile [21,22]. The BTC treatment algorithm, based on the latest European guidelines [23,24], is illustrated in Figure 1.

BTCs are characterized by heterogeneity in clinical presentation, anatomical location, tumor growth patterns, and molecular features [25,26,27]. This biological complexity contributes to poor responses to conventional treatments and justifies the significant efforts made to decipher the molecular landscape of these aggressive adenocarcinomas. Numerous genetic alterations with potential relevance for MTTs have been identified in BTCs, with certain mutations being more prevalent in specific subtypes. For example, small-duct iCCA frequently harbors actionable mutations, such as *IDH1/2* mutations and *FGFR2* fusions or rearrangements [28,29,30]. In contrast, *KRAS* and *SMAD4* mutations are more commonly associated with large duct iCCA and eCCA, while alterations in *HER2* and *CDKN2A/B* are frequently found in both eCCA and GBC [28,31,32].

Several MTTs are currently recommended by the European Society for Medical Oncology for the treatment of BTCs harboring specific genetic alterations [23]. These include: ivosidenib for tumors with *IDH1* mutations [30]; futibatinib or pemigatinib for *FGFR2* fusions or rearrangements [33,34]; dabrafenib in combination with trametinib for *BRAF^V600E^* mutations [35]; trastuzumab plus pertuzumab or zanidatamab for *HER2* overexpression and/or amplification [36,37]; entrectinib, larotrectinib, or repotrectinib for *NTRK* fusions [38,39,40]; and selpercatinib for *RET* fusions [41]. However, except for *IDH1* mutations, *FGFR2* fusions, and *HER2* alterations, the vast majority of BTC patients do not harbor these actionable mutations [28]. This highlights the urgent need for novel therapeutic strategies to reach a wider patient population.

Compared with other gastrointestinal (GI) malignancies, BTCs are associated with a relatively high tumor mutational burden (TMB), defined as the total number of somatic nonsynonymous mutations per megabase [42], making them promising candidates for immunotherapy, particularly with ICIs [43,44]. In recent years, combinations of immunotherapy with gemcitabine and cisplatin have been evaluated as first-line treatments for patients with locally advanced or metastatic BTC. Two pivotal phase III trials demonstrated significant survival benefits. The TOPAZ-1 trial showed that the addition of durvalumab to gemcitabine–cisplatin significantly improved OS (hazard ratio [HR]: 0.80; 95% confidence interval [CI]: 0.66–0.97; *p* = 0.021) [19]. Similarly, the KEYNOTE-966 trial demonstrated improved OS with the addition of pembrolizumab to gemcitabine–cisplatin (HR: 0.83; 95% CI: 0.72–0.95; *p* = 0.0034) [20]. Both regimens are now recommended as standard first-line therapies for advanced BTC [23]. Additionally, after the encouraging results of KEYNOTE-028 and KEYNOTE-158 trials [45,46], pembrolizumab is recommended for BTC patients with deficient mismatch repair (dMMR) and high microsatellite instability (MSI-H) who have not previously received immunotherapy. Approximately 1% of the tumors included in these trials were MSI-H, but the results for this population subset were not up to date. MMR is a component of the DNA damage repair (DDR) system, an essential set of cellular mechanisms for maintaining genomic stability and cell viability, and its deficiency can be easily detected in BTCs by immunohistochemistry (IHC) [47]. Interestingly, alterations in DDR genes are frequently observed in BTCs, representing another promising option for targeted therapeutic development [48].

Genomic instability is a hallmark of cancer resulting from the accumulation of DNA damage and/or dysfunctions of the DDR machinery [49]. In BTCs, chronic inflammation and congenital malformations of the biliary tree or gallbladder represent major risk factors [8,50,51]. Pro-inflammatory cytokines and nitric oxide contribute to DNA damage and can impair DDR pathways, thereby promoting BTC initiation and progression [52,53,54]. The increased expression of DDR proteins has been reported in patients with congenital biliary dilatation and has also been associated with BTC development [51]. According to the consensus statement of the European Network for the Study of CCA, DDR alterations may also play a role in chemoresistance [53]. In this review, we examine the frequency of alterations in DDR genes in each BTC subtype, explore their prognostic and predictive significance, and highlight current progress in therapeutic options targeting the DDR pathways.

## 2. DNA Damage Repair Mechanisms

**Figure 2:**
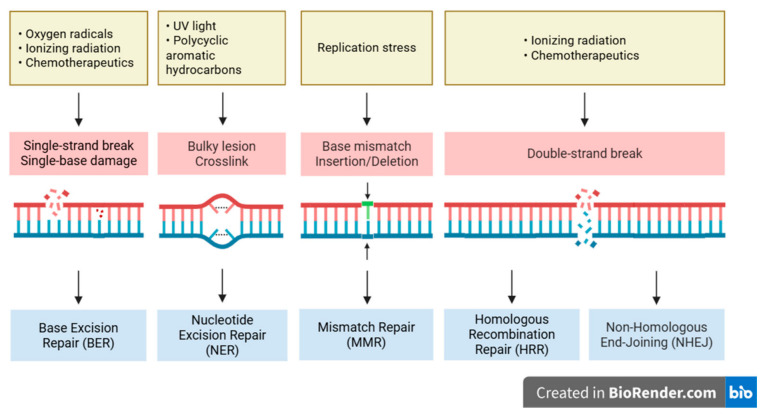
Summary of the major DNA damage causes (in yellow), types (in red), and repair mechanisms (in blue). Created in BioRender.com.

DDR mechanisms form an interconnected network of pathways that preserve the genomic integrity of human cells. These mechanisms detect DNA lesions, eliminate abnormal structures from the genome, and repair damaged sequences [55]. Distinct repair pathways are activated depending on the nature of the damage and whether it arises from endogenous or exogenous sources [54]. The main DDR pathways (Figure 2) include: (1) base excision repair (BER), which corrects single-strand breaks (SSBs) and single-base lesions induced by reactive oxygen species or ionizing radiation; (2) nucleotide excision repair (NER), which removes bulky DNA adducts and crosslinks caused by ultraviolet light or polycyclic aromatic hydrocarbons; (3) mismatch repair (MMR), which resolves replication-associated base mismatches, insertions, and deletions; and (4) homologous recombination repair (HRR) and non-homologous end-joining (NHEJ), which repair double-strand breaks (DSBs) resulting from chemotherapeutic agents or ionizing radiation [48,56,57]. The activity of DDR pathways is regulated by the coordinated expression of numerous genes. When canonical repair mechanisms are compromised, alternative pathways can compensate, such as single-strand annealing and alternative end-joining for the repair of DSBs [58,59]. All these repair processes are closely integrated with cell cycle checkpoints and survival pathways to ensure genomic stability throughout cell divisions [59].

## 3. DNA Damage Repair Alterations in Cancer

Alterations in DDR pathways are a common characteristic of many human cancers. Aberrations in genes encoding DNA damage signaling and repair proteins promote genomic instability which supports tumorigenesis, cancer progression, metastasis formation, and resistance to DNA-damaging therapies, such as chemotherapy and radiotherapy [54,59,60]. DDR alterations can emerge from somatic or germline mutations, but also from copy number alterations (CNAs), epigenetic changes, transcriptional dysregulation, altered protein stability, or perturbations in processes, such as DNA replication, chromatin remodeling, and nucleotide metabolism [54,59]. Importantly, not all DDR gene aberrations drive tumorigenesis; some represent passenger events [54]. When functional, these alterations can result in either deficiency or upregulation of DDR components.

Hopkins et al. classified DDR deficiencies in cancer into three classes [54]. First, defects in the repair of replication-associated DNA damage and DSBs commonly involve tumor suppressor genes, such as *BRCA1* and *BRCA2*, from the HRR machinery [61,62], as well as components of the NHEJ and *Fanconi anemia* pathways [63]. These alterations impair DSB repair, destabilize stalled replication forks, disrupt interstrand crosslink repair, and promote R-loop accumulation, contributing together to genomic instability [54,64]. Second, mutations affecting DNA damage signaling and checkpoint control genes, such as *ATM*, *ATR* (also involved in HRR), and their downstream effectors (e.g., *CHK1/2*), impair cell cycle arrest, apoptosis, and replication stress responses and cause further accumulation of genomic alterations [65,66,67,68]. Third, high mutational burden arising from defects in MMR (e.g., *MLH1*) [69], NER (e.g., *RPA*), BER (e.g., *MGMT*) genes, or proofreading-deficient DNA polymerases [70] lead to MSI and hypermutated tumor phenotypes [54,71].

Furthermore, the prevalence and biological significance of DDR alterations vary considerably across cancer types. For example, DDR alterations (except for MMR) are relatively uncommon in GI malignancies overall [72]. Clinically actionable alterations such as *BRCA2* (17%), *PALB2* (14%), *ATM* (11%), and *BRCA1* (8.6%) have been identified by next-generation sequencing (NGS) in a subset of 299 GI cancer cases, highlighting the need to better define their therapeutic importance in specific tumor contexts, including in BTCs [48,72]. Moreover, the interactions between the different branches of the DDR network may also differ between tumor types and may be altered by the microenvironmental context and treatments, also having potential significant therapeutic consequences [54].

## 4. DNA Damage Repair Alterations in Biliary Tract Cancers

BTCs remain aggressive malignancies with limited therapeutic options, and the prevalence and clinical significance of DDR alterations in this context are still not well-defined. Large-scale genomic studies, such as The Cancer Genome Atlas and the International Cancer Genome Consortium, have confirmed that CCA development is closely linked to recurrent alterations in DDR-related genes, such as *BRCA1*, *BRCA2*, *ATM*, *ATR*, *BAP1*, *RAD51*, or *MLH1* [73,74]. DDR alterations, such as *BRCA2* and *ATM* mutations, were also identified in patients diagnosed with GBC [75,76]. In BTCs, DDR alterations are mostly somatic. Patients with *Lynch* syndrome (characterized by dMMR) or with germline mutations of *BAP1* or *BRCA1/2* genes are uncommon, but they have a higher risk of developing BTC [77,78,79,80,81]. DDR alterations in BTC can also be due to CNAs, gene expression dysregulation, or epigenetic alterations [76,82]. However, the reported frequencies vary considerably, ranging approximately from 25% to 70% [57,83,84,85]. These variations are explained by differences in cohort composition, tumor subtype, sequencing approach (whole-genome vs. whole-exome vs. targeted sequencing), and the variable definitions of DDR alterations as discussed earlier [43,53,84,86,87]. The prevalence of DDR alterations in BTC subtypes is indicated in Table 1.

Different patterns of DDR gene alterations have been identified in BTC. In a cohort of 422 FFPE samples from BTC patients, higher mutation rates and higher TMB (≥20 mut/Mb) were observed in eCCA and GBC compared with iCCA [88]. Weinberg et al. later reported that 5.8% of GBC, 3.5% of iCCA, and 2% of eCCA cases had a high TMB (≥17 mut/Mb) [89]. A larger study including 803 BTC patients demonstrated later that germline mutations occurred primarily in DDR genes and less than 5% of the tumors were hypermutated (≥9.36 mut/Mb). Interestingly, the overall median TMB was low (1.23 mut/Mb) although almost 40% of iCCA patients showed a high TMB [74]. Furthermore, according to the data from several studies, high TMB was found to be correlated with alterations in DDR genes [85,90,91].

Molecular profiling in advanced BTC is currently an essential step, but it remains technically challenging due to insufficient tumor content in a significant proportion of tissue samples. This limitation may be partially overcome by approaches based on circulating tumor DNA and cell-free DNA [23,86]. More specifically, in a large NGS study of 1292 cases, a global prevalence of 3.6% of BTCs carrying *BRCA* mutations has been reported. *BRCA2* mutations were more frequent than *BRCA1* mutations in several anatomical subtypes, and these *BRCA*-mutated tumors were associated with higher TMBs and increased rates of MSI-H/dMMR, suggesting a recurrent molecular profile with therapeutic potential [92]. However, HRR deficiency is not limited to *BRCA1/2* alone; it also includes other genes and “*BRCAness*” (or “*BRCA*-like”) phenotypes, characterizing *BRCA*-wild-type tumors that behave similarly to *BRCA*-mutated tumors, such as those with *BAP1* loss. There is currently no international consensus on which alterations or assays should define DDR alterations in BTC, complicating patient management [93].

**Table 1:** Frequencies, types, and clinical significance of DDR alterations in the different BTC subtypes.

DDR Mechanisms and Genes		Alteration Rate (%)				Alteration Type (Functional Impact)	Therapeutic/Clinical Significance (Level of Evidence)	References
		iCCA	pCCA/ dCCA	GBC	All BTCs			
Base excision repair (BER)	*MGMT*	38	26–60	59–62	38	Promoter methylation (gene inactivation)	Clinical: Poor prognosis (meta-analysis).	[82,94,95,96]
	*PARP*	NA	NA	NA	~1–2	Missense and substitution/indel SM (loss)	In vitro/clinical: PARP inhibitors are the most advanced DDR-targeting therapies in BTC (NCT03207347, NCT03337087, NCT03639935, NCT03878095, NCT04042831, NCT04306367). Results of NCT03212274 (olaparib), NCT03991832 (olaparib + durvalumab), NCT04298021 (ceralasertib + olaparib), NCT04779151 (dostarlimab + niraparib), NCT05222971 (olaparib ± durvalumab), NCT06441747 (olaparib + durvalumab) and NCT07269158 (durvalumab/pembrolizumab ± venadaparib) trials are pending.	[74,97,98,99,100,101,102,103,104,105,106,107,108,109]
Mismatch repair (MMR)	All MMR genes	5	4–65.7	51.3–59	11–14	SM, GM (dMMR)	Clinical: MSI-H/dMMR patients sensitive to pembrolizumab (SoC).	[23,45,81,85,94,110,111]
Non-homologous end-joining (NHEJ)	*ARID1A*	19–22	14	16.4	5.4–21.7	SM, gene deletion (loss)	Clinical: Potential marker of worse prognosis and higher risk of recurrence in iCCA.	[76,81,86,111,112,113]
Homologous recombination repair (HRR)	*ATM*	4–9	5–8.57	5.6–6.3	4.5–11.8	Missense, nonsense, and substitution/indel SM, truncation, GM (loss)	In vitro: ATM inhibition (AZD0156) effective in BTC cell lines (monotherapy/combination regimens).	[28,74,76,86,90,91,112,113,114,115,116,117]
	*ATR*	NA	NA	~1.3	3–6	Missense and substitution/indel SM, truncation (loss)	In vitro: ATR inhibition (AZD6738) effective in BTC cell lines (monotherapy/combination regimens). Clinical: ORR of 0% after treatment of CCA patients with *IDH* mutation with olaparib and ceralasertib (NCT03878095). Results of NCT04298021 (ceralasertib + durvalumab) and NCT04491942 (elimusertib + cisplatin ± gemcitabine) trials are pending.	[74,76,81,102,112,118,119,120,121]
	*ATRX*	NA	NA	NA	1.8–4	SM (loss)	NA	[86,112]
	*BAP1*	1–50	~4–50	~1.3–9.5	4–8.8	Missense SM, rearrangement, GM (loss)	Clinical: Potential marker of worse prognosis; mCCA patient with *BAP1* mutation derived benefit from olaparib (case report).	[74,76,81,86,91,112,113,117,122,123,124,125,126,127,128,129]
	*BARD1*	NA	NA	NA	2.5	SM, GM (loss)	NA	[74,112]
	*BLM*	NA	NA	NA	~1–1.9	Missense and substitution/indel SM (loss)	NA	[74,112]
	*BRCA1*	0.4–9.23	~1–8	0.3–4	0.9–1.9	Missense and substitution/indel SM, GM (loss)	Clinical: Sensitivity to PARP inhibitors (SoC).	[23,28,74,86,90,92,112,117,130]
	*BRCA2*	2.7–20	~2–8	4	3.3–4.4	Missense, nonsense and indel SM, GM (loss)	Clinical: Sensitivity to PARP inhibitors (SoC).	[23,28,74,76,90,92,117,130,131]
	*CHK1*	NA	NA	NA	~0.5–1	Missense and nonsense SM (loss)	In vitro/in vivo: Treatment with rabusertib (CHK1 inhibitor) was effective against *KRAS*-mutated iCCA cells and was associated with a significant decrease in PARP1 levels. Clinical: Combination of prexasertib (CHK1/2 inhibitor) with standard treatments showed acceptable safety profiles in the phase I NCT02124148 trial.	[74,112,132]
	*CHK2*	NA	NA	NA	~0.3–1.9	Missense and substitution/indel SM, GM (loss)	Clinical: Combination of prexasertib (CHK1/2 inhibitor) with standard treatments showed acceptable safety profiles in the phase I NCT02124148 trial.	[74,86,112]
	*FANC*	NA	NA	~2	2.5–7.3	Missense and nonsense SM, CNA, rearrangement, GM (loss or upregulation)	In vitro/clinical: Potential association of upregulation of *FANC* genes with resistance to gemcitabine.	[76,112,133]
	*NBN*	NA	NA	NA	~1–1.4	Missense SM (loss)	NA	[74,112]
	*PALB2*	12.31	NA	~1.3	1.9	Missense, nonsense and substitution/indel SM, GM (loss)	Clinical: Sensitivity to PARP inhibitors (SoC).	[23,74,76,90,112,134]
	*PBRM1*	9.9	4–4.5	~5–7.5	5–21	Missense, nonsense and substitution/indel SM (loss)	Clinical: *PBRM1* mutations may sensitize BTC tumors to DDR (e.g., PARP, ATR) inhibition.	[74,76,81,113,117,135,136]
	*RAD50*	NA	NA	NA	~1.3–1.8	Missense SM, GM (loss)	NA	[74,112]
	*RAD51 (B,C,D)*	NA	NA	NA	0.8–4.6	Missense SM, translocation (CNA), GM (loss)	In vitro: RAD51 inhibitor enhanced sensitivity of BTC cells to gemcitabine/cisplatin.	[74,112,133]
	*WRN*	NA	NA	NA	4.9	SM (loss)	NA	[112]
	All HRR genes	NA	NA	NA	5–43	SM/GM/CNA (mostly HRR deficiency)	Clinical: Sensitivity to PARP inhibitors.	[44,85,112]

*ARID1A: AT-rich interaction domain 1A; ATM: ataxia telangiectasia mutated; ATR: ataxia telangiectasia and Rad3-related protein; ATRX: alpha thalassemia/intellectual disability syndrome X-linked; BAP1: BRCA1-associated protein 1; BARD1: BRCA1-associated RING domain 1; BLM: Bloom syndrome protein; BRCA1: breast cancer 1; BRCA2: breast cancer 2;* BTC: biliary tract cancer; *CHK1: checkpoint kinase 1; CHK2: checkpoint kinase 2;* CNA: copy number alteration; dCCA: distal cholangiocarcinoma; *FANC: Fanconi anemia complementation group;* GBC: gallbladder carcinoma; GM: germline mutation; iCCA: intrahepatic cholangiocarcinoma; mCCA: metastatic cholangiocarcinoma; *MGMT: O^6^-methylguanine-DNA methyltransferase;* NA: not available; *NBN: nibrin; PALB2: partner and localizer of BRCA2; PARP: poly-(ADP-ribose) polymerase; PBRM1: polybromo-1;* pCCA: perihilar cholangiocarcinoma; *RAD50: radiation sensitive 50; RAD51: radiation sensitive 51 (paralogs B, C, and D);* SM: somatic mutation; SoC: standard of care; *WRN: Werner syndrome ATP-dependent helicase*.

## 5. DNA Damage Repair Alterations as Biomarkers

### 5.1. DNA Damage Repair Alterations as Prognostic Biomarkers

DDR alterations may have prognostic significance in GI malignancies according to published data, including BTCs. In colorectal cancer, ATM- or BRCA-deficient tumors were associated with dMMR and improved survival [137]. In BTCs, genomic analyses of 412 samples identified germline alterations in DDR genes (e.g., *BRCA1/2*, *MLH1*, *MSH2*, and *RAD51D*) in approximately 11% of patients. Common mutations in genes such as *TP53*, *KRAS*, *ARID1A*, and *ATR* were reported, and patients with *ARID1A*-mutated tumors were independently associated with worse prognosis [81].

In multicenter cohorts, patients with *MGMT* promoter hypermethylation and with lower *MGMT* mRNA and protein expressions were associated with significantly shorter OS [82,94]. Another meta-analysis across several solid tumors confirmed that the prognostic impact of *MGMT* epigenetic inactivation is tumor-specific, especially in BTCs (HR = 2.31) [95]. Furthermore, in a cohort of 229 CCA samples, Wang et al. evaluated the prognostic relevance of single nucleotide polymorphisms in several DDR genes and found that the TT genotype of *XPC rs2228001* (involved in NER) was significantly associated with improved survival [138]. In GBC specifically, deficiency of SMARCA4, a tumor suppressor involved indirectly in DDR and chromatin-remodeling complexes, was associated with MMR proficiency, reduced OS and progression-free survival (PFS) [139]. Although these studies suggest that *ARID1A* and *MGMT* alterations could represent potential prognostic biomarkers of BTC, prospective validation remains necessary.

### 5.2. DNA Damage Repair Alterations as Predictive Biomarkers for Immunotherapy Response

BTCs are characterized by an inflamed and immunosuppressive tumor microenvironment comprising cancer-associated fibroblasts, tumor-associated macrophages, myeloid-derived suppressor cells, and dysfunctional T and NK cells. These cells release cytokines, such as IL-6, TNF-α, and TGF-β, to induce immune escape [43,140]. Upregulation of immune checkpoint molecules, including PD-1/PD-L1 and CTLA-4, has been reported in BTCs and is associated with worse prognosis [44,141], justifying the potential use of ICIs [43]. In parallel, genomic studies have demonstrated that DDR defects increase tumor immunogenicity by promoting genomic instability, neoantigen generation, and recognition of cancer cells by the immune system [142,143]. However, few studies have assessed the predictive relevance of DDR alterations for response to ICIs [144].

MSI-H/dMMR tumors have been shown to be sensitive to pembrolizumab, leading to the first Food and Drug Administration approval [45,48] and making this biological status the most established predictive biomarker for anti-PD-1 antibody response [145]. In BTC specifically, although MSI-H/dMMR accounts for less than 5% of cases [146], the KEYNOTE-158 study reported an objective response rate (ORR) of approximately 40% in this subgroup, and better survival outcomes [46,110]. Interestingly, a case report involving a patient with recurrent pCCA demonstrated that combined immunotherapy induced a shift from microsatellite stable/proficient MMR status to an MSI-H/dMMR tumor profile, emphasizing the clinical impact of treatment-induced changes in tumor biology [147]. High TMB is also a promising biomarker according to data from KEYNOTE-158 (≥10 mut/Mb) and DDR-mutated cohorts treated with nivolumab (≥12 mut/Mb), but its predictive impact has not been consistent in combination regimens or in unselected patients [43,46,144,148].

ICI monotherapy appears to be more effective in biomarker-enriched populations with advanced BTC. In KEYNOTE-028, an ORR of ~13% with short PFS and OS were reported after pembrolizumab treatment of PD-L1-positive patients [46]. Nivolumab demonstrated moderate response rates in phase I/II studies and better outcomes were observed in selected subsets [144,149,150]. Other agents, such as durvalumab and bintrafusp alfa, have shown acceptable safety profiles but did not show significant efficacy in second-line settings [151,152]. Therefore, immunotherapy and chemotherapy combination strategies have gained importance in BTCs, as chemotherapy may improve immune responses by increasing neoantigen release and modulating the tumor immune microenvironment [43,153]. The phase III TOPAZ-1 and KEYNOTE-966 trials demonstrated that durvalumab or pembrolizumab plus gemcitabine–cisplatin significantly improved OS and PFS compared with chemotherapy alone in the first-line setting, establishing a new SoC [19,20]. Alternative combinations that may additionally enhance tumor immunogenicity, including ICIs with anti-angiogenic agents, PARP inhibitors (PARPis), radiotherapies, and locoregional therapies, are also under investigation [43,48,54]. Despite these advances, response rates remain lower than in highly immunogenic tumors. The identification of robust and independent predictive biomarkers beyond MSI-H/dMMR remains an urgent priority since the combined positive score (based on the PD-L1 expression assessed by IHC) does not effectively stratify the patients that could respond to pembrolizumab.

### 5.3. DNA Damage Repair Alterations as Predictive Biomarkers for Chemotherapy Response

Platinum compounds (e.g., cisplatin, oxaliplatin) and alkylating agents (e.g., temozolomide, cyclophosphamide) are commonly used DNA-damaging agents in oncology [48,58]. In ovarian cancer, cisplatin has shown its efficacy due to the high frequency of HRR deficiency in patients [54]. In advanced BTCs, gemcitabine–cisplatin remains an important component of standard first-line treatment, highlighting the potential platinum sensitivity of these malignancies. Platinum agents induce cytotoxicity through DNA damage, and emerging genomic data suggest that DDR alterations may influence their efficacy [48,154,155,156].

External evidence from pancreatic and colorectal cancers has shown that HRR defects were associated with improved outcomes following platinum-based chemotherapy, supporting the predictive potential of DDR status [157,158]. Multiple retrospective genomic studies have revealed that BTC patients with DDR alterations, often associated with higher TMB and HRR deficiency [85,91], showed improved outcomes after first-line platinum-based chemotherapy, with significantly longer PFS and OS [83,86,91]. More recent data further reinforce this concept, as in a cohort of 180 BTC patients, deleterious DDR alterations were independently associated with improved ORR, PFS, and OS in those treated with platinum-based chemotherapy as well as in those treated with the combination of immunotherapy and chemotherapy [131].

Tumor cells can also acquire increased DNA repair ability, thereby playing a pivotal role in chemoresistance [159,160,161]. For instance, Dicer (involved in RNA processing) levels are increased after DNA damage by chemotherapy, thereby activating of the NHEJ pathway in colon cancer [162]. More specifically, ERCC1 (involved in NER) and XRCC1 (involved in BER) nuclear expression might influence clinical outcomes of BTC patients following adjuvant gemcitabine treatment [163]. This observation was later confirmed in patients with advanced BTC, where high ERCC1 expression was associated with shorter OS and PFS [164]. An additional study reported that p53R2 overexpression (involved in DDR) is associated with gemcitabine resistance in CCA cell lines [165]. A recent study mentioned that the overexpression of complement component 1q subcomponent-binding protein (C1QBP, a membrane protein involved in DDR) is associated with decreased sensitivity to platinum-based treatments and with CCA proliferation [166]. In GBC cells, elevated XRCC1 expression levels are associated with resistance to 5-FU [167], while Src and DUSP1 (both indirectly involved in DDR) upregulations reduce cisplatin-induced DNA damage [168,169].

### 5.4. DNA Damage Repair Alterations as Predictive Biomarkers for Radiotherapy Response

Somatic and germline mutations in DDR genes may increase tumor sensitivity to chemotherapy but also to radiotherapy by preventing cancer cells from repairing treatment-induced DNA damage [48,137]. There is currently no validated predictive biomarker for radiotherapy response in BTC. However, similar to other solid tumors, radiotherapy may be used in the adjuvant setting and/or for unresectable disease, providing a strong rationale for combining radiation with DDR-targeting agents [87,170,171,172,173]. For example, inhibition of ATR amplifies the radiation-induced inflammatory tumor microenvironment and DNA damage, forcing irradiated cells to enter mitosis with unrepaired DSBs [174,175]. These combinations can be extended to other HRR and NHEJ components involved in DSB repair.

Furthermore, emerging evidence suggests that combining radiotherapy with immunotherapy could be more effective [176,177,178], by promoting neoantigen release and modulating the tumor immune microenvironment to improve immune response, even in unselected BTC patients [43,179]. Interestingly, patients with low TMB, MMR proficiency, microsatellite stability, and low PD-L1 expression—subgroups generally considered less responsive to ICIs—showed potential benefit from radioimmunotherapy [180]. DDR inhibition may also potentiate the effects of radioimmunotherapy, supporting a combination strategy that could potentially overcome treatment resistance in BTCs.

## 6. DNA Damage Repair Proteins as Therapeutic Targets

Treatments such as chemotherapy and radiotherapy induce DNA damage, but pharmacological DDR inhibition aims to exploit the genomic instability of cancer cells in a more selective manner. Targeting DDR mechanisms is a promising anticancer strategy based on the idea that amplifying the pre-existing genetic instability of tumor cells can push them to replication failure, mitotic catastrophe, and cell death by apoptosis [48,54]. This strategy, known as synthetic lethality, involves inhibiting key DDR mediators [181] and is particularly relevant in BTCs, which frequently harbor alterations in DDR pathways [85].

PARPis are currently the most clinically advanced DDR-targeting drugs in cancer treatment [182]. PARP1/2 are key enzymes in SSB repair through BER and also contribute to DSB repair through HRR and NHEJ [183]. PARPis block BER by trapping PARP on DNA and lead to lethal replication-associated DSBs in HRR-deficient tumors [184]. In BTCs, several PARPis, including olaparib, rucaparib, niraparib, veliparib, and talazoparib, have been and are still under in vitro, in vivo, and clinical evaluation as monotherapies or in combination with chemotherapy, immunotherapy, and targeted therapies [97,98,101,103,105,185], especially in patients with *BRCA1/2*, *PALB2*, *BAP1*, or other HRR gene alterations [48,87,112,129,186,187,188]. Nevertheless, the molecular heterogeneity of BTCs and resistance mechanisms such as *BRCA* reversion mutations or replication fork stabilization can reduce the efficacy of PARPi treatment [189,190].

Several other DDR targets have also been investigated for the treatment of *BRCA*-wild-type tumors. ATR, ATM, WEE1, and CHK1/2 inhibitors represent attractive DDR-targeting agents, particularly in tumors with genomic instability or *ATM* loss [99,116,191]. *ATR* inhibitors, such as ceralasertib and berzosertib, have shown encouraging safety and efficacy results, especially when combined with PARPis or chemotherapy, in in vitro BTC models and in preliminary clinical trials including BTC patients [119,192,193,194]. The combination of PARPis and ATM inhibitors has been shown to lead to cell cycle arrest and enhanced apoptosis in CCA in vitro models [115]. More recently, the combination of an ATM inhibitor with DNA-damaging agents (cisplatin or photon irradiation) was shown to selectively eliminate gemcitabine-resistant iCCA cells, characterized by DNA ligase I (involved in alternative end-joining) downregulation [114]. WEE1 inhibitors (e.g., adavosertib) force tumor cells into premature mitotic entry despite presence of DNA damage and have demonstrated promising effect in combination regimens, including in *BRCA*-mutated iCCA [118,195,196]. CHK1/2 inhibitors are still under clinical development in BTC, but they may further sensitize tumors to PARPis, chemotherapy, or radiotherapy [197,198]. Alternatively, another study demonstrated that cholesterol depletion by lovastatin enhanced the sensitivity of GBC cells to cisplatin through the inactivation of CHK1, CHK2, and γ-H2AX [199].

Further potential targets include *DNA-PK*, *PLK1*, *POLθ*, *RAD51*, *RAD52*, *USP1*, and *WRN*, as well as other mediators of alternative repair pathways and additional components that may be combined with DDR inhibitors [54,87]. DNA-PK inhibitors, such as peposertib combined with radiotherapy and immunotherapy, are currently being examined in hepatobiliary malignancies [200]. In iCCA specifically, He et al. showed that targeting xanthine oxidoreductase-mediated EGFR stabilization leads to increased DDR impairment and enhanced sensitivity to EGFR tyrosine kinase inhibitors [201]. In parallel, chromatin-remodeling impairments caused by *BAP1* or *PBRM1* mutations may sensitize tumors to PARP and ATR inhibition [135]. Nanoparticle-based chemotherapy delivery systems are also being explored to improve tumor selectivity and therapeutic efficacy in *PBRM1*-deficient BTCs [136]. Additionally, recent efforts were made to synthesize new chemotherapeutic compounds called “Aurkines”, which selectively induce DSB and apoptosis of treatment-naïve and cisplatin-resistant CCA tumors without harming normal cholangiocytes [202].

As stated previously, a major future direction of BTC management involves combining DDR inhibitors with immunotherapy. DDR defects impair genomic integrity, increase neoantigen load, and activate innate immune pathways such as *cGAS-STING*, which can convert “cold” tumors into “hot” ones, characterized by higher immune infiltrate [203,204]. Early-phase clinical data suggest that the combination of PARPis and ICIs may enhance antitumor immunity, even in *BRCA*-wild-type populations [205]. Encouraging results from two phase II studies show that olaparib and ICI combination after platinum-based chemotherapy leads to antitumor activity and provides clinical benefit in patients with HRR-deficient and metastatic pancreatic cancer [206,207]. Furthermore, ATR inhibition can modulate PD-L1 expression and enhance tumor sensitivity to T-cell-mediated cytotoxicity, supporting combination approaches [208]. Table 2 summarizes the previous and latest clinical trials related to DDR-targeted treatments in BTCs.

## 7. Conclusions and Future Directions

**Figure 3:**
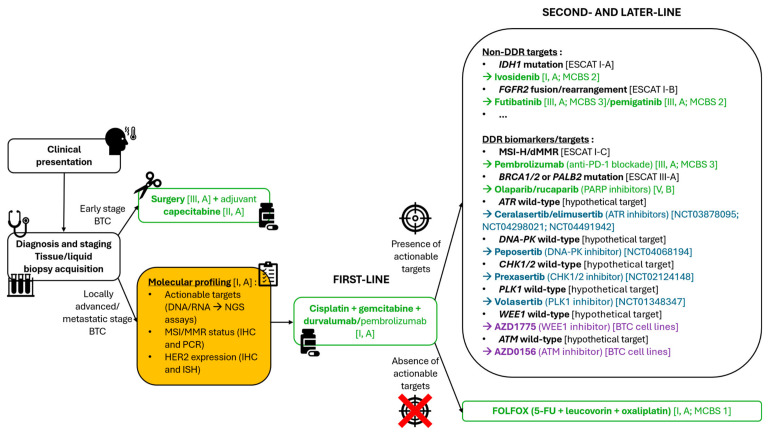
Clinical decision-making algorithm from BTC diagnosis to established and investigational DDR-targeted therapies. The recommendations of the European Society of Medical Oncology (ESMO) were classified according to their level of evidence and their grade, and where applicable, with the ESMO-MCBS v1.1 (ESMO—Magnitude of Clinical Benefit Scale). ESCAT (ESMO Scale for Clinical Actionability of molecular Targets) scores of genomic alterations represent the level of evidence as biomarkers for using targeted therapies. Green: standard treatments; blue: investigational treatments currently under clinical evaluation; and purple: treatments tested in in vitro models. 5-FU: 5-fluorouracil; *ATM: ataxia telangiectasia mutated; ATR: ataxia telangiectasia and Rad3-related protein; BRCA1/2*: *breast cancer 1 or 2*; BTC: biliary tract cancer; *CHK1/2: checkpoint kinase 1/2;* DDR: DNA damage repair; *DNA-PK: DNA-dependent protein kinase*; *FGFR2*: *fibroblast growth factor receptor 2*; HER2: human epidermal growth factor receptor 2; *IDH1*: *isocitrate dehydrogenase 1*; IHC: immunohistochemistry; ISH: in situ hybridization; MSI-H/dMMR: high microsatellite instability and deficient mismatch repair; NGS: next-generation sequencing; *PALB2*: *partner and localizer of BRCA2;* PCR: polymerase chain reaction; *PLK1: polo-like kinase 1*. Adapted from [23].

BTCs are a heterogeneous group of aggressive GI malignancies associated with very poor prognoses and limited effective therapeutic options. Alterations in DDR pathways are observed in ~25% to ~70% of patients and contribute to tumorigenesis, disease progression, and treatment response. Although relatively uncommon, several potential and established DDR-related prognostic (e.g., *MGMT* inactivation) and/or predictive (e.g., MSI-H/dMMR, *BRCA1/2* mutations) biomarkers have been described in the literature, highlighting the importance of DDR alterations in the management of BTC patients. Some DDR defects may paradoxically represent actionable therapeutic targets through synthetic lethality strategies; combinations of DDR inhibitors; or combinations with chemotherapy, radiotherapy, and immunotherapy. These approaches have shown promising results in both in vitro and in vivo models, and several clinical trials are ongoing to extend the population of patients who may benefit from these DDR-based targeted therapies (Figure 3).

Nevertheless, the low frequency of actionable DDR alterations and the various resistance mechanisms are challenges that must be considered. In the future, it will be necessary to identify novel actionable DDR targets and liquid biopsies that could be of great help in cases of insufficient tumor tissue. It will also be important to pursue large-scale, prospective, subtype-specific, and biomarker-guided trials to assess the predictive and prognostic value of DDR alterations, as most of the studies presented in this review are retrospective and include heterogeneous BTC subtypes. Other indicators, such as *BAP1* loss, HRR deficiency scores, RAD51 foci formation, or other DDR gene signatures, should also be considered for patient selection. Furthermore, future research should integrate the complex crosstalk of TMB, HRR deficiency, tumor microenvironments, and immune responses in these challenging malignancies.

## Figures and Tables

**Table 2:** Clinical trials related to DDR inhibitors in BTCs (ClinicalTrials.gov accessed on 26 May 2026). Adapted from [87].

Study Identifier (Status, Location)	Phase	Treatments	Targets	N	Conditions	Primary Objectives and Time Frame	Results	Ref.
NCT01348347 (Completed; Japan)	I	Volasertib	PLK1	15	Advanced lung, skin, esophageal, gastric, pancreatic, and hepatobiliary tumors	Number of participants with DLT and MTD up to 21 days	Number of participants with DLT: 200 mg cohort: 0/3 300 mg cohort: 0/6 350 mg cohort: 2/6 MTD: 300 mg	[209]
NCT02124148 (Completed; USA)	I	Prexasertib + cisplatin (A) or cetuximab (B) or pemetrexed (C) or fluorouracil (D) or LY3023414 (E)	CHK1/2 DNA-PK	167	Advanced solid cancers, including CCA	MTD up to 24 weeks for each combination	(A): 80 mg/m^2^ (B): 70 mg/m^2^ (C): NA (D): 40 mg/m^2^ (E): NA	[198]
NCT03207347 (Completed; USA)	II	Niraparib	PARP	37	Mesothelioma; uveal melanoma; renal cell carcinoma; CCA (DDR-wild-type vs. DDR-mutated)	ORR at 1 year	DDR-wild-type cohort: 1/18 (5.6%) DDR-mutated cohort: 0/13 (0%)	[99]
NCT03212274 (Active, not recruiting; USA)	II	Olaparib	PARP	89	*IDH*-mutant advanced/recurrent solid neoplasm; glioma; glioblastoma; CCA	ORR up to 8 weeks	NA	[210]
NCT03337087 (Unknown status; USA)	I/II	Rucaparib + irinotecan + 5-FU ± leucovorin calcium	PARP	18	Metastatic GI malignancies, including mBTC	Number of participants with DLT up to 28 days	1/12 (8.3%)	[100]
NCT03639935 (Completed; USA)	II	Rucaparib + nivolumab	PARP PD-1	32	mBTC	Proportion of patients alive and without radiological or clinical progression at 4 months	17/31 (54.8%)	[101]
NCT03878095 (Active, not recruiting; USA)	II	Olaparib + ceralasertib	PARP ATR	24	*IDH*-mutant solid tumors, including CCA	ORR up to 30 days	0/24 (0%)	[102]
NCT03991832 (Recruiting; Canada)	II	Olaparib + durvalumab	PARP PD-L1	58	*IDH*-mutant solid tumors, including glioma and CCA	ORR and DCR at 3 years	NA	[103]
NCT04042831 (Active, not recruiting; USA)	II	Olaparib	PARP	32	mBTC with DDR alterations	Number of patients alive and progression-free survival at 8 weeks	23/31 (74.2%)	[104]
NCT04068194 (Active, not recruiting; USA)	I/II	Avelumab ± peposertib (+hypofractionated radiotherapy)	PD-L1 DNA-PK	103	Advanced/metastatic solid tumors and hepatobiliary malignancies	MTD up to 28 days and ORR at 12 weeks	NA	[200]
NCT04298021 (Unknown status; South Korea)	II	Ceralasertib + durvalumab; ceralasertib + olaparib	ATR PD-L1 PARP	74	aBTC	DCR at ~1 year	NA	[120]
NCT04306367 (Completed; USA)	II	Olaparib + pembrolizumab	PARP PD-1	14	aCCA	ORR up to 2 years	2/13 (15.4%)	[105]
NCT04491942 (Active, not recruiting; USA)	I	Elimusertib + cisplatin ± gemcitabine	ATR	74	Advanced solid tumors, including BTC	Incidence of adverse events up to 28 days (after treatment completion) and recommended phase II dose of elimusertib up to 21 days (from treatment start date)	NA	[121]
NCT04779151 (Terminated; France)	II	Dostarlimab + niraparib	PD-1 PARP	51	Renal cell carcinoma; head and neck cancer; urothelial bladder cancer, and GI cancer, including BTC	ORR at 15 weeks	NA	[106]
NCT05222971 (Recruiting; South Korea)	II	Olaparib ± durvalumab	PARP PD-L1	62	mBTC	Six-month PFS rate	NA	[107]
NCT06441747 (Recruiting; Australia)	II	Olaparib + durvalumab	PARP PD-L1	40	aCCA	Efficacy of PARP and PD-L1 inhibition 12 months post randomization	NA	[108]
NCT07269158 (Not yet recruiting; South Korea)	I/II	Durvalumab/ pembrolizumab ± venadaparib	PD-L1 PD-1 PARP	160	aBTC	Recommended phase II dose and PFS up to 4 years	NA	[109]

5-FU: 5-fluorouracil; aBTC: advanced biliary tract cancer; aCCA: advanced cholangiocarcinoma; ATR: ataxia telangiectasia and Rad3-related protein; BTC: biliary tract cancer; CCA: cholangiocarcinoma; CHK1/2: checkpoint kinase 1/2; DCR: disease control rate; DDR: DNA damage repair; DLT: dose limiting toxicity; DNA-PK: DNA-dependent protein kinase; GI: gastrointestinal; *IDH: isocitrate dehydrogenase*; mBTC: metastatic biliary tract cancer; MTD: maximum tolerated dose; NA: not available; ORR: overall response rate; PARP: poly-(ADP-ribose) polymerase; PD-1: programmed cell death protein 1; PD-L1: programmed cell death ligand 1; PFS: progression-free survival; PLK1: polo-like kinase 1.

## Data Availability

No new data were created or analyzed in this study. Data sharing is not applicable to this article.
